# Social determinants of male partner attendance in women’s prevention-of mother-to-child transmission program in Malawi

**DOI:** 10.1186/s12889-020-09800-4

**Published:** 2020-11-30

**Authors:** Isotta Triulzi, Olivia Keiser, Claire Somerville, Sangwani Salimu, Fausto Ciccacci, Ilaria Palla, Jean Baptiste Sagno, Jane Gondwe, Cristina Marazzi, Stefano Orlando, Leonardo Palombi, Giuseppe Turchetti

**Affiliations:** 1 Institute of Management, Scuola Superiore Sant’Anna, Piazza Martiri Libertà, 56127 Pisa, Italy; 2 Institute of Global Health, University of Geneva, Geneva, Switzerland; 3 Gender Center, Graduate Institute of International and Development Studies, Geneva, Switzerland; 4 DREAM Programme, Community of Sant’Egidio, Blantyre, Malawi; 5 UniCamillus, Saint Camillus International University of Health Sciences, Rome, Italy; 6 Lumsa University, Rome, Italy; 7 Department of Biomedicine, University of Tor Vergata, Rome, Italy

**Keywords:** Knowledge, HIV, Gender, Access, Social determinants, Behaviour, Pregnancy

## Abstract

**Background:**

Male partners are rarely present during PMTCT (Prevention-Mother-To-Child-Transmission) services in Sub-Saharan Africa (SSA). Male involvement is increasingly recognised as an important element of women’s access to care. This study aims to identify the socio-demographic characteristics, HIV-Knowledge, Attitude and Practice (KAP) among women accompanied and not accompanied by their male partners.

**Methods:**

We included pregnant women enrolled in PMTCT programme between August 2018 and November 2019 in the Southern Region of Malawi. Eligible women were aged 18 years or older, living with a male partner, enrolled for the first time in one of the four selected facilities. We provided a KAP survey to women and their partners attending the facilities. Our primary objective was to assess and analyse the proportion of women who were accompanied by their partner at least once. We applied descriptive statistics and logistic regressions to study the association between being accompanied and explanatory variables.

**Results:**

We enrolled 128 HIV-positive women: 82 (64.1%) were accompanied by their male partners and 46 (35.9%) were alone. In the multivariable model, women’s unemployment and owning a means of transport are negatively associated with male attendance (respectively adjusted OR 0.32 [95% CI, 0.11–0.82] and 0.23 [95% CI, 0.07–0.77]), whereas, in the univariable model, high women’s level of knowledge of HIV is positively associated with male attendance (OR 2.17 [95% CI, 1.03–4.58]). Level of attitude and practice toward HIV were not significantly associated to our study variable.

**Conclusions:**

Our study shows a high male attendance in Malawi compared to other studies performed in SSA. This study highlights that women’s level of knowledge on HIV and their economic condition (employment and owning a means of transport) affects male attendance. Moreover, the study points out that gender power relationships and stringent gender norms play a crucial role thus they should be considered to enhance male involvement.

**Supplementary Information:**

The online version contains supplementary material available at 10.1186/s12889-020-09800-4.

## Background

In 2018, 37.9 million people across the globe were living with HIV/AIDS. Of these, 36.2 million were adults and 1.7 million were children (< 15 years old) [1]. Worldwide, there were 1.3 million pregnant women living with HIV in 2018, among them the 82% received Antiretroviral Therapy (ART) for Prevention Mother-To-Child Transmission (PMTCT) [2]. The WHO African Region was most severely affected with 25.7 million people HIV positive people [3].

Malawi is one of the countries with higher HIV prevalence in the adult population (15–49 years) amounting to 9.2% in 2018. It is estimated that one million Malawians, adults and children, are infected with HIV. Women infected represent 59.8% of adults living with HIV [4].

Previous literature shows that in Sub-Saharan Africa (SSA), male partners are scarcely present during PMTCT services [5]. In many low-income countries, the involvement of men in maternal services is increasingly recognised as an important element of women’s access to needed care [6–8]. Male involvement is not a well-defined concept and currently there is no single widely used indicator to measure it. Several definitions have been used in previous studies [9–12], but the most comprehensive one was proposed by Muwanguzi et al. [13]. In this study male involvement in PMTCT was not limited to accompanying the woman to the clinic and perform Couple HIV Testing and Counselling (CHTC), but also supporting the woman during the treatment from an economic and psychosocial point of view. However, in some circumstances, men involvement could be considered disadvantageous to their partners since it may reinforce their role and promote men control over women’s decision [9, 10]. Some studies have demonstrated that male support is relevant to women’s adherence to therapy in PMTCT. The study performed by Katirayi et al. [14] in Zimbabwe and Malawi for instance shows that one of the main barriers to women initiating and adhering to ART were their male partners. Zacharius et al. [15] showed that women with male support in PMTCT were 3.5 times more likely to have good adherence than those without support. Male involvement has been recognized as a priority in PMTCT but currently remains a challenge in most low- and middle-income countries. Few programs have been implemented, but much improvement is still needed to enhance the male involvement, thus it is important to identify better strategies in PMTCT and in healthcare services in general [16].

This paper represents the first stage of a study aiming to identify interventions to improve male involvement along the cascade of HIV services in Malawi. This paper aims to: 1) assess the attendance of male partners in PMTCT; 2) describe the socio-demographic characteristics of women enrolled in PMTCT, accompanied by their male partner or not; 3) determine the level of knowledge, attitude and practice toward HIV/AIDS associated with male attendance; 4) study the association between being accompanied by a male partner and socio-demographic characteristics and knowledge attitude and practice on HIV/AIDS.

## Methods

### Study population and setting

We included in the study pregnant women enrolled in Malawi’s national PMTCT programme between 10 August 2018 and 30 November 2019 at four health facilities in the Southern region of Malawi. These health facilities are located in Blantyre (urban), Machinjiri (peri-urban), Chileka (peri-urban) and Balaka (rural) and they are part of the DREAM (Disease Relied through Excellent and Advanced Means) programme, a public health program runs by the Community of Sant’Egidio in 11 African Countries (sub recipient of Global Fund). This program focuses on HIV/AIDS, TB and NCDs [17–19].

The pregnant women were eligible if they were aged 18 years or older, lived with a male partner, enrolled for the first time in a DREAM facility and willing to sign a written informed consent. We have decided to include only women living with a male partner, assuming that a cohabitant partner could impact more on their behaviours.

This study is part of larger research program that aims to investigate the impact of male participation on women’s ART adherence in PMTCT in Malawi.

We have administered a Knowledge, Attitude and Practices (KAP) survey [20] to women and their male partners attending the health facilities. KAP surveys assess knowledge gaps, cultural beliefs, and behavior of people with HIV/AIDS. It identifies misconceptions or misunderstandings that may represent barriers to access the health care, to uptake of interventions and to improve adherence to ART. We have designed the survey questionnaire following the guidelines for conducting a KAP survey [20, 21]. Previous literature has been taking into account in the questionnaire designing phase and to analyse the responses [22–24]. The final questionnaire consisted of 27 questions or statements (see Additional file 1,2,3): 15 on knowledge of HIV transmission (1–8), prevention and anti-retroviral therapy (9–15), 7 on attitudes towards HIV/AIDS, and 5 on sexual, alcohol or drug use behaviour. The answer to each question had three possible answers (yes, no, do not know).

To better adapt the questions to the Malawian context, we discussed the readability and clarity of the questionnaire with two healthcare workers and a medical doctor. We then pilot-tested it in a small group of randomly selected women (*n* = 22). The KAP survey was translated in the local language and administered to a woman and her male partner by community health workers of DREAM facility.

If the couple went together to the first visit, the survey was administered separately to the women and their partners. If the woman was alone, the healthcare worker would give her the invitation card (recommended by Minister of Health in Malawi) asking to go with the partner to the next visit. During the second visit, the healthcare worker would interview the woman (KAP survey). If the male partner did not attend any clinical visit during the 6 weeks after the first one, the questionnaire was to be completed only by the woman. If the male partner did attend, he has been interviewed, and this survey was not included in the paper due to small sample size. It is recommended that women have at least three PMTC visits after the first one. All the surveys administered to the women were included in the analysis. We adopted a convenience sampling method since this is a pilot study.

### Outcomes

Male attendance to health facilities at least once in the 6 weeks follow-up period was the primary outcome. Among the not-accompanied women, we collected secondary male partner specific outcomes such as the proportion of women who i) disclosed their status to their male partner, ii) reported to their male partners that they had a clinical appointment, iii) asked the male partners’ permission before going to the facilities, iv) delivered the invitation slip and v) the transport fee was provided by their partners.

In addition to the KAP survey we also used the data from the electronic medical record system on the women enrolled in the study. These data included age, educational level, type of job, number of people in the family, socio-economic condition (e.g. availability of electricity), owning a means of transport, means of transport to get to facility and travel time from household to facility. A woman was considered lost to follow-up (LTFU) when she missed to collect the doses of antiretroviral drugs for more than 2 months.

### Statistical analysis

The characteristics of the women were described using Fisher test or chi- square tests for categorical variables, and Wilcoxon rank sum tests for continuous variables.

To determinate the level of knowledge, attitude and practice of respondents, we asked women to answer “yes”, “no” or “do not know” to every questions. Following literature [22, 23, 25] a score of ‘1’ was assigned for each correct answer and ‘0’ for each wrong answer. As it was previously done by other researchers [22–27], overall knowledge was determined by aggregating correct answers from all questions according to other researchers. The maximum attainable score was 15 and the minimum score was 0. For the seven attitude related questions, each positive response was assigned a score of ‘1’, and each negative response a score of ‘0’. In the same manner, for the five practice related questions, each safe response was assigned a score of ‘1’, and each risky response as a score of ‘0’. We defined the overall level of attitude and practice by aggregating positive attitude answers and safe practise answers. The scoring range of the attitude section was from 0 to 7 and of the practice section was from 0 to 5. Then, we determined the percentage. Based on previous research [23, 24], levels of knowledge, attitude and practice were categorized in two segments depending on their median score, as data were not normally distributed. Low and high level of knowledge, positive and negative attitude and safe and risky practice presented the two categories. To evaluate how our KAP scoring method impacted the analysis of results, we also run the analysis considering an alternative scoring system: score of ‘1’ for each correct, ‘0’ for each uncertain, ‘-1’ for each wrong answers.

We compared the single answer score using Mann-Whitney tests and the overall level of knowledge, attitude and practice using Mann-Whitney tests with 95% confidence intervals (CI) in order to determine if there was any difference in knowledge, attitude and practice among women accompanied and not accompanied by their male partner.

We conducted univariable logistic regressions to study the association between male attendance (yes, no) and explanatory variables. In the univariable logistic regression the explanatory variables considered age, education (no education/primary school, secondary school/pre-University), employment (employed, unemployed), owning a means of transport such as cars, motor cycles and bikes (yes, no), means of transport to get to facility (minibus, motorbike, car or bike, by foot, other), time to reach the facility (0–89 min, > 89 min), economic condition (availability of electricity in household, yes, no), and knowledge (low, high), attitude (positive, negative), practice (safe, risky) toward HIV. We included in the multivariable model our variables of interest (knowledge, attitude and practice) and all the variables that resulted to be significantly associated with male attendance in the univariable logistic regression. The “unemployed” category includes houseworkers (stay-at-home) and unemployed women, the “employed” category includes employee (formal job with contract) and temporary job (informal job with no contract).

We imputed missing values of explanatory variables using multiple imputation with chained equations (MICE). To improve the imputation we added the following variables [28]: mother alive (yes/no), owns a phone (yes/no), gestational age (1–3, 4–6, > 7), piped water available in the dwelling (yes/no), owned means of transport (bike, motorbike, car, bus, others). Furthermore, we considered our outcome in the imputation. We ran the model on 20 imputed datasets for each analysis and combined the estimates with Rubin’s rule [29].

All analysis were performed using STATA 13.

## Results

### Characteristics of study participant and KAP survey

We screened 142 HIV-positive pregnant women. A total of 128 women met the eligibility criteria: 82 (64.1%) were accompanied by their male partners and 46 (35.9%) were alone. Two women came with their partners before they were transferred to another facility. None of them were lost to follow-up during the follow-up period.

Table 1 shows the socio-demographic characteristics of the participants, and their knowledge, attitude and practice toward HIV among the accompanied and non-accompanied women. The median age of the women was 27.8 years (IQR 22.8–32.3) with no significant difference in the two groups. Half of the women had no education or attended the primary school (46.8%). Significant differences in terms of employment and owning a means of transport were found among the two groups: among the accompanied women, 76.9% were employed, and only 6% of them had a means of transport. Among non-accompanied, 60.9% were employed and 23.9% had a transport. In term of economic condition, size of family, type of means of transport and travel time to the healthcare facility no significant differences were observed in the two group.

**Table 1:** Socio-demographic information of HIV-positive women enrolled in this study in Malawi (*n* = 128)

	All (***n*** = 128)	Women accompanied by the male partners (***n*** = 82)	Women not accompanied by the male partner (***n*** = 46)	***P***-value^**a**^
**Socio-demographics variables**				
**Age** Median (IQR)	27.8 (22.8–32.3)	27.4 (22.0–32.3)	28.6 (24.6–32.3)	0.356
**Educational level**				0.823
No education/ Primary	60 (46.9)	39 (47.6)	21 (45.6)	
Secondary/Pre-University	65 (50.8)	41 (50.0)	24 (52.2)	
Missing	3 (2.3)	2 (2.4)	1 (2.2)	
**Healthcare center location**				0.302
Machinjiri	74 (57.8)	36 (43.9)	38 (82.6)	
Chileka	35 (27.3)	31 (37.8)	4 (8.6)	
Balaka	12 (9.4)	10 (12.2)	2 (4.4)	
Blantyre	7 (5.5)	5 (6.1)	2 (4.4)	
**Employment Status**				**0.031**
Employed	91 (71.1)	63 (76.9)	28 (60.9)	
Unemployed	31 (24.2)	14 (17.0)	17 (37.0)	
Missing	6 (4.7)	5 (6.1)	1 (2.1)	
**Partner’s Employment**				0.359
Employed	45 (35.2)	25 (30.5)	20 (43.5)	
Unemployed	70 (54.7)	48 (58.5)	22 (47.8)	
Missing	13 (10.1)	9 (11.0)	4 (8.7)	
**Size of your family**				0.280
0–3	31 (24.2)	23 (28.0)	8 (17.4)	
> 3	96 (75.0)	59 (72.0)	37 (80.4)	
Missing	1 (0.8)	0(0)	1 (2.2)	
**Travel time to the facility (minutes)**				0.151
0–89 min	92 (71.9)	55 (67.1)	37 (80.4)	
> 89 min	34 (26.5)	26 (31.7)	8 (17.4)	
Missing	2 (1.6)	1 (1.2)	1 (2.2)	
**Owning a means of transport**				**0.003**
No	109 (85.2)	75 (91.5)	34 (73.9)	
Yes	16 (12.5)	5 (6.0)	11 (23.9)	
Missing	3 (2.3)	2 (2.5)	1 (2.2)	
**Means of transport**				0.494
Minibus, motorbike	115 (89.8)	74 (90.3)	41 (89.1)	
Bike, by foot, other	11 (8.6)	6 (7.3)	5 (10.9)	
Missing	2 (1.6)	2 (2.4)	0 (0)	
**Electricity available in the dwelling**				0.167
Yes	77 (60.2)	29 (35.4)	22 (47.8)	
No	51 (39.8)	53 (64.6)	24 (52.2)	
**Knowledge, attitude and practice toward HIV**				
**Knowledge**				**0.040**
Low	68 (53.1)	38 (46.3)	30 (65.2)	
High	60 (46.9)	44 (53.7)	16 (34.8)	
**Attitude**				0.214
Negative	52 (40.6)	30 (36.6)	22 (47.8)	
Positive	76 (59.4)	52 (63.4)	24 (52.2)	
**Practice**				0.836
Risky	68 (53.1)	43 (52.4)	25 (54.3)	
Safe	60 (46.9)	39 (47.6)	21 (45.7)	
**Total**	128 (100.0)	82 (100.0)	46 (100.0)	

^a^ Chi-square or Wilcoxon rank sum test

Responses to questions on the KAP survey are reported in the Additional files (1,2,3). The 53.7% of women accompanied by male partner showed a high level of knowledge on HIV/AIDS; only the 34.8% of women not accompanied presented high level of knowledge. No significant difference between the two groups was observed in term of median level of knowledge, attitude and practice (Additional file 4).

Table 2 shows “male partner specific” variables among the women who attended the facility alone.

**Table 2:** “Male partner specific” variables among the non-accompanied women (*n* = 46)

Variable	N of women, (%)
Disclose HIV status to partner	45 (97.8)
Report of having an appointment to partner	44 (95.7)
Ask Partner’s permission to partner	42 (95.4)
Receive Transport refund to partner	42 (95.4)
Deliver the invitation slip to partner	43 (93.5)
Partner is tested	29 (63.0)
Partner is HIV positive	24 (52.2)

As shown in Table 2, 29 (63.0%) partners of non-accompanied women took a HIV test and 24 (52.2%) tested positive. Their median age was 35.5 years (IQR 32–38) and 20 (43.5%) were employed.

In the univariable analysis, several sociodemographic variables were associated with male attendance (Table 3). KAP survey analysis showed that male attendance was associated to woman’s level of knowledge on HIV/AIDS. High women’s level of knowledge was associated with higher likelihood of male attendance (OR 2.17; 95% CI, 1.03–4.58), whereas women’s unemployment and owning a means of transport were associated with lower likelihood of male partners’ participation (respectively OR 0.36 [95% CI, 0.16–0.84] and OR 0.21 [95% CI, 0.07–0.64]).

**Table 3:** Univariable and multivariable analysis examining association between male attendance and explanatory variables (*n* = 128)

	Crude OR	Adjusted OR
	Estimate (95% CI)	Estimate (95% CI)
**Socio-demographic variables**		
Age (years)	0.97 (0.91–1.03)	0.97 (0.89–1.04)
Education		
No education/primary	1	1
Secondary/Pre-University	0.92 (0.44–1.91)	0.87 (0.38–1.97)
Employment		
Employed	1	**1**
Unemployed	**0.36 (0.16–0.84)**	**0.32 (0.11–0.82)**
Owning a means of transport		
No	1	1
Yes	**0.21 (0.07–0.64)**	**0.23 (0.07–0.77)**
Travel time to the facility (minutes)		
0–89	1	
> 89	2.28 (0.93–5.55)	
Electricity available in the dwelling		
No	1	
Yes	0.60 (0.29–1.24)	
Means of transport		
Minibus, motorbike, car	1	
Bike, by foot, other	0.67 (0.19–2.34)	
**Knowledge, attitude and practice toward HIV**		
Level of Knowledge		
Low	**1**	1
High	**2.17 (1.03–4.58)**	2.31 (0.91–5.88)
Level of Attitude		
Negative	1	1
Positive	1.59 (0.76–3.30)	1.02 (0.40–2.57)
Level of Practice		
Risky	1	1
Safe	1.08 (0.52–2.23)	1.01 (0.45–2.29)

*OR* odds ratio, *CI* confidence interval

In the multivariable model, unemployment and owning a mean of transport remained negatively associated with male attendance (respectively adjusted OR 0.32 [95% CI, 0.11–0.82] and 0.23 [95% CI, 0.07–0.77]). Attitude and practice did not show any significant effect (respectively adjusted OR 1.02 [95% CI 0.40–2.57] and OR 1.01 [95% CI 0.45–2.29]).

We ran the analysis adopting an alternative KAP scoring system and we confirmed the results previously described, except for women’s knowledge that was no more associated with attendance in the univariable analysis (Additional file 5, 6).

## Discussion

In this study we investigated the male partner involvement in a sample of women included in a PMTCT program in Malawi. We evaluated the association between the male attendance in care and the socio-demographic characteristics, HIV-knowledge, attitude and practice related to HIV/AIDS. To our knowledge this is the first study comparing the socio-economic characteristics and knowledge, attitudes and practices of women accompanied and not accompanied to PMTCT service by their partners.

Several studies evaluate the male involvement. Our study shows that the male attendance is 64.1% in PMTCT. This is comparable to the result reported by Rosenberg et al. [30] in a unblinded randomised controlled trial comparing the adoption of the invitation slip versus the use of invitation slip plus tracing (52% vs 74%) in Lilongwe, Malawi. Other studies performed in Malawi reported that the male attendance at antenatal clinic were: 13.7% in a retrospective study performed in 2004–2006 in Mwanza District [11]; 10.7% in a observational study in Bwaila Hospital in Lilongwe in 2009 [31]; 19% in a randomized control trial where the use of an invitation slip was compared to non-intervention [32]. Other studies reported male attendance rates of 16% in Uganda [9], 35% in South Africa [33], 36% in Kenya [34], and 53.5% in Tanzania [35]. Our result shows a good male attendance compared to other studies. One reason could be that in the last few years more attention and more interventions have targeted men in the country.

Our study shows that being unemployed and owing a means of transport are negatively associated with male attendance. These results reveal the role of the social determinants of health [36]. Owning a means of transport may indicate easier access to the healthcare facilities and higher economic conditions. However, among the women who possess a means of transport, 13 women reach the clinic by minibus, 2 by foot and 1 by other means of transport. Thus, owning a means of transport does not imply to use your own vehicle to reach the clinic. Jennings et al. [37] observed that more empowered women (with higher economic status and access to a means of transport) are significantly less likely to have their partner’s presence at facilities in Malawi. This finding could support our result if we consider owning a mean of transport as a proxy of women’s empowerment. Thus, women highly empowered in healthcare and in household decisions show less need to invite men. One interpretation of the relation between unemployment and male attendance could be that unemployed woman reaches the healthcare facility alone because man needs to work during the day, whereas woman has more time available to spend to get to the healthcare centre [8]. In fact, married women (72%) are less likely to have been employed in the last 12 months than currently married men (98%) and women are more likely to being paid less for their work compared to men in Malawi, as reported by DHS [38]. This result could reflect complex gender roles in the Malawian social context where men are the providers of the family and women are primary carers and responsible for activities associated with care of the home and family. Thus, some women view male accompaniment as a foreign concept, and they do not want men to invade their territory [39, 40]. Gender roles and norms strongly affect the male attendance.

Moreover, financial restraints could reduce ability to spend on transport fee which in turn result in reduced male partner attendance; providing the travel expense on public transport for two people can be challenging in this context [41, 42] . In fact, distance to healthcare facility is still indicated by 56% of women as a key barrier to health access when they are sick [43] with a median travel time of 1 h [44]. In regards of this, in the future it would be interesting to consider the transport fee spent to get to the facility, the geo-distribution [45] of the population and distance to facilities in our sample in order to see if any association exists with male attendance. Moreover, the country is extremely poor: 51.5% of the population income fell below the poverty line in 2016 [46]. A recent published research showed that 56% of household would spend less than 1 US$ to reach the healthcare facility and that in the country are present only 14.3 cars and motor cycles per 1000 people [47]. In fact, lack of financial resources to pay for transport cost to reach a hospital has been reported by 39% of male and 59% of female head of households as one of the main barriers [44].

A relevant result is that a higher score in the level of HIV-knowledge was associated with attendance of the male partner in the univariate analysis that could reflect the fact that women with high level of knowledge may be more likely to voice and more capable to negotiate and involve their partners in PMTCT. The study of Ampt et al. [48] reported that male involvement in Maternal and Newborn Health was positively associated with women’s level of education and men’s level of knowledge on MNH in Myanmar. On the contrary, this paper shows that women’s education does not play a significant role on male attendance. This finding highlights that women may be a better negotiator for the support they need, despite their educational level. However, the association was no more significant in the multivariate model being borderline.

This study has some limitations. Firstly, the small sample size prevented stratifications in statistical analysis and reduced the power of some analysis. Secondly, KAP questionnaire was surveyor-assisted incurring in social desirability bias [49]. Thirdly, there are no standards questions in KAP questionnaire that allow us to compare our results with others in a similar context. Although, the majority of KAP questions are also present in the Demographic and Health Surveys (DHS) performed in several Sub-Saharan African countries by USAIDS [50], a direct comparison was not possible due to the different sampling method. Fourthly, we included in this study only women living with a male partner. As additional limitation, in our study distance to facility was not collected as part of information required to participants, despite it could play a role with respect to the likelihood of men involvement.

Our study suggests that male partners attendance could be improved by considering other socio-determinants of health beyond women’s health education; public health initiatives may consider to target the couples or the family identity and the overall health of the relationship rather than solely women rights and emancipation. Interventions enabling to identify norms of male support within the couple without compromise the women’s autonomy and promote men’s control over women are strongly recommended. Moreover, male involvement would be enhanced with integrated multi-component strategies [51] enabling to deliver healthcare services closer to the villages or at home and to incentive male partners (economic or non-economic incentive) [52, 53]. As shown by Salmen et al. [54] promoting social network engagement through the development of “microclinic” intervention in villages (consisting of a small network of 5–15 neighbours, relatives and friends) may be promising and could be designed to evaluate the involvement of male partners. In order to design effective intervention, we have to recognise wider Malawian gender orders and gender norms around masculinities [55]. Such “restrictive gender norms” [56] and the wider societal inequality regimens [57] are reproduced in the healthcare systems [58] and may impact male engagement. Hay et al. [56] showed that gender norms and inequities are determined and reinforced in families, communities, structures and policies and perpetuated by institutions including healthcare systems. Previous studies in Malawi suggest that gender norms and masculinities [42] are factors in access, attitudes and stigma [55, 59]. Gender inequalities in health system could be disrupted” from within, through actions for reform and transformation, and from the outside, through progressive policies and laws and community pressure and activism” [56] involving a multi-disciplinary team of public health experts. Further studies to understand why and how men in Malawi become involved are required. Intersectional studies considering Malawian gender roles and norms, masculinities, income and healthcare system inequality including geographical accessibility and means of transport [60, 61] are needed in order to implement effective interventions to increase the involvement of male partners [62] and to achieve Sustainable Development Goals and universal health coverage [63].

## Conclusions

The results of this study show a higher level of male attendance in Malawi compared to other studies conducted in Sub-Saharan Africa. Women’s knowledge on HIV seems to play an important role in the male attendance in PMTCT. Owning a means of transport and being unemployed are two key factors negatively affecting attendance. These factors are strictly linked to the hierarchical nature of gender relations within the household and gender norms. Addressing socio-determinants of health as gender and socio-economic conditions is of paramount importance to improve the health of the entire family.

## Supplementary Information

**Additional file 1. **Knowledge on HIV/AIDS transmission (1–8), prevention and treatment (9–15) among women accompanied (*n* = 82) and not accompanied by the male partner (*n* = 46).

**Additional file 2.** Positive attitude toward people living with HIV/AIDS among women accompanied (n = 82) and not accompanied by the male partner (n = 46).

**Additional file 3. **Safe practice toward HIV/AIDS among women accompanied (*N* = 82) and not accompanied by the male partner (n = 46).

**Additional file 4. **Median of correct knowledge, positive attitude and safe practice toward HIV/AIDS among women accompanied (*n* = 82) and not accompanied by the male partner (*n* = 46).

**Additional file 5 **Knowledge, attitude and practice toward HIV of women in PMTCT in Malawi (*n* = 128)*. *A different scoring system was applied (score of ‘1’ for each correct, ‘0’ for each uncertain, ‘-1’ for each wrong answers).

**Additional file 6.** Univariable and multivariable analysis examining association between male attendance and explanatory variables (n = 128)*. *A different scoring system was applied (score of ‘1’ for each correct, ‘0’ for each uncertain, ‘-1’ for each wrong answers).

## Data Availability

The datasets used and/or analysed during the current study available from the corresponding author on reasonable request.

## Abbreviations

HIV: Human immunodeficiency virus
AIDS: Acquired immune deficiency syndrome
DREAM: Disease relied through excellent and advanced means
PMTCT: Prevention mother to child transmission
CHTC: Couple HIV testing and counselling
ART: Antiretroviral therapy
SSA: Sub-Saharan Africa
LTFU: Lost to follow-up

## References

[1] Global HIV and AIDS statistics | Avert. Available from: https://www.avert.org/global-hiv-and-aids-statistics. [cited 2020 Jul 24].

[2] WHO | Prevention of mother-to-child transmission (PMTCT). Available from: https://www.who.int/gho/hiv/epidemic_response/PMTCT_text/en/. [cited 2020 Jul 24].

[3] HIV/AIDS | WHO | Regional Office for Africa. Available from: https://www.afro.who.int/health-topics/hivaids. [cited 2020 Jul 24].

[4] United Nation AIDS. Malawi | UNAIDS. Available from: https://www.unaids.org/en/regionscountries/countries/malawi. [cited 2020 Apr 6].

[5] World Health Organization (WHO). Male involvement in the prevention of mother-to-child transmission of HIV. Geneva: WHO Library Cataloguing-in-Publication Data Male; 2012.

[6] World Health Organization (2015). WHO recommendations for maternal and interventions on health promotion newborn health 2015. Vol. 151.

[7] Tokhi M, Comrie-Thomson L, Davis J, Portela A, Chersich M, Luchters S (2018). Involving men to improve maternal and newborn health: a systematic review of the effectiveness of interventions. Plos One.

[8] Phiri N, Tal K, Somerville C, Msukwa MT, Keiser O (2019). “I do all i can but i still fail them”: health system barriers to providing option B+ to pregnant and lactating women in Malawi. Plos One.

[9] Byamugisha R, Astrom AN, Ndeezi G, Karamagi CAS, Tylleskar T, Tumwine JK (2011). Male partner antenatal attendance and HIV testing in eastern Uganda: a randomized facility-based intervention trial. J Int AIDS Soc.

[10] Farquhar C, Kiarie JN, Richardson BA, Kabura MN, John FN, Nduati RW (2004). Antenatal couple counseling increases uptake of interventions to prevent HIV-1 transmission. J Acquir Immune Defic Syndr.

[11] Kalembo FW, Zgambo M, Mulaga AN, Yukai D, Ahmed NI (2013). Association between male partner involvement and the uptake of prevention of mother-to-child transmission of HIV (PMTCT) interventions in Mwanza District, Malawi: a retrospective cohort study. Plos One.

[12] Hampanda K, Abuogi L, Musoke P, Onono M, Helova A, Bukusi E (2020). Development of a novel scale to measure male partner involvement in the prevention of mother-to-child transmission of HIV in Kenya. AIDS Behav.

[13] Muwanguzi PA, Nassuna LK, Voss JG, Kigozi J, Muganzi A, Ngabirano TD (2019). Towards a definition of male partner involvement in the prevention of mother-to-child transmission of HIV in Uganda: a pragmatic grounded theory approach. BMC Health Serv Res.

[14] Katirayi L, Chadambuka A, Muchedzi A, Ahimbisibwe A, Musarandega R, Woelk G (2017). Echoes of old HIV paradigms: reassessing the problem of engaging men in HIV testing and treatment through women’s perspectives. Reprod Health.

[15] Zacharius KM, Basinda N, Marwa K, Mtui EH, Kalolo A, Kapesa A (2019). Low adherence to option B + antiretroviral therapy among pregnant women and lactating mothers in eastern Tanzania. Plos One.

[16] WHO Regional Office for Africa. Implementation of Option B+ for Prevention of Mother-To-Child Transmission of HIV: the Malawi Experience. Brazzaville: WHO/AFRO Library Cataloguing–in–Publication; 2014.

[17] Ristin MMC, De Luca S, Palombi L, Scarcella P, Ciccacci F, Ceffa S (2014). Predictors of adverse outcomes in HIV-1-infected children receiving combination antiretroviral treatment: results from a DREAM cohort in sub-Saharan Africa. Pediatr Infect Dis J.

[18] Orlando S, Triulzi I, Ciccacci F, Palla I, Palombi L, Marazzi MC (2018). Delayed diagnosis and treatment of tuberculosis in HIV+ patients in Mozambique: a cost-effectiveness analysis of screening protocols based on four symptom screening, smear microscopy, urine LAM test and Xpert MTB/RIF. Plos One.

[19] Ciccacci F, Tolno VT, Doro Altan AM, Liotta G, Orlando S, Mancinelli S (2019). Noncommunicable diseases burden and risk factors in a cohort of hiv+ elderly patients in Malawi. AIDS Res Hum Retrovir.

[20] World Health Organization (WHO). Advocacy, communication and social mobilization for TB control: a guide to developing knowledge, attitude and practice surveys. WHO/HTM/STB/2008.46. Geneva: WHO Library Cataloguing-in-Publication Data Advocacy; 2008.

[21] World Health Organization (WHO). Knowledge, Attitude, and Practices (KAP) survey during cholera vaccination campaighs: Guidance for Oral Cholera Vaccine Stockpile Campaigns. Geneva: Working Group on Monitoring & Evaluation; 2014.

[22] Shokoohi M, Karamouzian M, Mirzazadeh A (2016). HIV Knowledge , Attitudes , and Practices of Young People in Iran : Findings of a National Population-Based Survey in 2013. Plos One.

[23] Thanavanh B, Kasuya H, Sakamoto J (2013). Knowledge , attitudes and practices regarding HIV / AIDS among male high school students in Lao People ’ s Democratic Republic. BMC Public Health.

[24] Nubed CK, Akoachere JFTK (2016). Knowledge, attitudes and practices regarding HIV/AIDS among senior secondary school students in Fako Division, South West Region, Cameroon. BMC Public Health.

[25] Fatema K, Hossain S, Natasha K, Chowdhury HA, Akter J, Khan T (2017). Knowledge attitude and practice regarding diabetes mellitus among nondiabetic and diabetic study participants in Bangladesh. BMC Public Health.

[26] Sakr S, Ghaddar A, Hamam B, Sheet I. Antibiotic use and resistance: an unprecedented assessment of university students’ knowledge, attitude and practices (KAP) in Lebanon. BMC Public Health. 2020;20:535. 10.1186/s12889-020-08676-8 PMC7169022 32306940

[27] Ul Haq N, Hassali MA, Shafie AA, Saleem F, Farooqui M, Haseeb A (2013). A cross-sectional assessment of knowledge, attitude and practice among hepatitis-B patients in Quetta, Pakistan. BMC Public Health.

[28] JAC S, White IR, Carlin JB, Spratt M, Royston P, Kenward MG (2009). Multiple imputation for missing data in epidemiological and clinical research: Potential Pitfalls. BMJ.

[29] Rubin D (2004). Multiple imputation for nonresponse in surveys.

[30] Rosenberg NE, Mtande TK, Saidi F, Stanley C, Jere E, Paile L (2015). Recruiting male partners for couple HIV testing and counselling in Malawi’s option B+ programme: an unblinded randomised controlled trial. Lancet HIV.

[31] Mphonda SM, Rosenberg NE, Kamanga E, Mofolo I, Boa E, Mwale M (2014). Assessment of peer-based and structural strategies for increasing male participation in an antenatal setting in Lilongwe, Malawi. Afr J Reprod Heal.

[32] Nyondo AL, Choko AT, Chimwaza AF, Muula AS (2015). Invitation cards during pregnancy enhance male partner involvement in prevention of mother to child transmission (PMTCT) of human immunodeficiency virus (HIV) in Blantyre, Malawi: a randomized controlled open label trial. Plos One.

[33] Mohlala BKF, Boily MC, Gregson S (2011). The forgotten half of the equation: randomized controlled trial of a male invitation to attend couple voluntary counselling and testing. AIDS..

[34] Osoti AO, John-Stewart G, Kiarie J, Richardson B, Kinuthia J, Krakowiak D (2014). Home visits during pregnancy enhance male partner HIV counselling and testing in Kenya: a randomized clinical trial. AIDS..

[35] Jefferys LF, Nchimbi P, Mbezi P, Sewangi J, Theuring S (2015). Official invitation letters to promote male partner attendance and couple voluntary HIV counselling and testing in antenatal care: an implementation study in Mbeya Region, Tanzania. Reprod Health.

[36] Schulz A, Northridge ME (2004). Social determinants of health: implications for environmental health promotion. Heal Educ Behav.

[37] Jennings L, Na M, Cherewick M, Hindin M, Mullany B, Ahmed S (2014). Women’s empowerment and male involvement in antenatal care: analyses of demographic and health surveys (DHS) in selected African countries. BMC Pregnancy Childbirth..

[38] National Statistical Office (NSO) [Malawi] I. Malawi Demographic and Health Survey 2015–16. 2017.

[39] Flax VL, Yourkavitch J, Okello ES, Kadzandira J, Katahoire AR, Munthali AC (2017). “If my husband leaves me, I will go home and suffer, so better cling to him and hide this thing”: The influence of gender on Option B+ prevention of mother-to-child transmission participation in Malawi and Uganda. Anglewicz P, editor. Plos One.

[40] Kululanga LI, Sundby J, Malata A, Chirwa E (2012). Male involvement in maternity health care in Malawi. Afr J Reprod Health.

[41] Flax VL, Yourkavitch J, Okello ES, Kadzandira J, Katahoire AR, Munthali AC (2017). “If my husband leaves me, I will go home and suffer, so better cling to him and hide this thing”: The influence of gender on Option B+ prevention of mother-to-child transmission participation in Malawi and Uganda. Plos One.

[42] Nyirenda L, Makwiza I, Bongololo G, Theobald S (2006). A gender perspective on HIV treatment in Malawi: a multi-method approach. Source Gend Dev.

[43] Health Care System- Malawi (2016). Minister of Health and Population, Republic of Malawi.

[44] Varela C, Young S, Mkandawire N, Groen RS, Banza L, Viste A. Transportation barriers to access health care for surgical conditions in Malawi a cross sectional nationwide household survey. BMC Public Health. 2019;19:264. 10.1186/s12889-019-6577-8 PMC6402149 30836995

[45] Maina J, Ouma PO, Macharia PM, Alegana VA, Mitto B, Fall IS (2019). A spatial database of health facilities managed by the public health sector in sub Saharan Africa. Sci data.

[46] The World Bank In Malawi - Malawi Overview. World Bank. 2019. Available from: https://www.worldbank.org/en/country/malawi/overview#1. [cited 2020 Apr 10].

[47] Ministry of Transport and Public Works. Malawi Goverment. Lilongwe; 2019.

[48] Ampt F, Mon MM, Than KK, Khin MM, Agius PA, Morgan C (2015). Correlates of male involvement in maternal and newborn health: a cross-sectional study of men in a peri-urban region of Myanmar. BMC Pregnancy Childbirth.

[49] Launiala A. How much can a KAP survey tell us about people’s knowledge, attitudes and practices? Some observations from medical anthropology research on malaria in pregnancy in Malawi. Anthropol Matters J. 2009;11(1):1–13.

[50] United Stated Agency International Development USAID. The DHS Program - Quality information to plan, monitor and improve population, health, and nutrition programs. Available from: https://dhsprogram.com/. [cited 2020 Apr 6].

[51] Triulzi I, Palla I, Ciccacci F, Orlando S, Palombi L, Turchetti G. The effectiveness of interventions to involve men living with HIV positive pregnant women in low-income countries: A systematic review of the literature. BMC Health Serv Res. 2019;19:943. 10.1186/s12913-019-4689-6 PMC6902537 31815620

[52] USAID Office of Population and Reproductive Health Bureau for Global Health. Essential Considerations for Engaging Men and Boys for Improved Family Planning Outcomes; 2018. Available at: https://www.usaid.gov/sites/default/files/documents/1864/Engaging-men-boys-family-planning-508.pdf.

[53] Besada D, Rohde S, Goga A, Raphaely N, Daviaud E, Ramokolo V (2016). Strategies to improve male involvement in PMTCT option B plus in four African countries: a qualitative rapid appraisal. Glob Health Action.

[54] Salmen CR, Hickey MD, Fiorella KJ, Omollo D, Ouma G, Zoughbie D (2015). “Wan Kanyakla” (we are together): community transformations in Kenya following a social network intervention for HIV care. Soc Sci Med.

[55] Chikovore J, Hart G, Kumwenda M, Chipungu GA, Corbett L (2015). Control, struggle, and emergent masculinities: a qualitative study of men’s care-seeking determinants for chronic cough and tuberculosis symptoms in Blantyre, Malawi. Glob Health Action.

[56] Hay K, McDougal L, Percival V, Henry S, Klugman J, Wurie H (2019). Disrupting gender norms in health systems: making the case for change. Lancet..

[57] Acker J (2006). Inequality regimes gender, class, and race in organizations. Source Gend Soc.

[58] Kanter RM, Stein B. Life in organizations : workplaces as people experience them. University of Michigan: Basic Books; 1979. p. 444.

[59] Moynihan C (1998). Theories of masculinity the biological concept of sex-a positivist approach. BMJ..

[60] Hankivsky O (2012). Women’s health, men’s health, and gender and health: implications of intersectionality. Soc Sci Med.

[61] Somerville C (2020). Why global health can offer more on gender. BMJ Glob Heal.

[62] Manda-Taylor L, Mwale D, Phiri T, Walsh A, Matthews A, Brugha R (2017). Changing times? Gender roles and relationships in maternal, newborn and child health in Malawi. BMC Pregnancy Childbirth..

[63] Comrie-Thomson L, Tokhi M, Ampt F, Portela N, Hersich M, Khanna E (2015). Challenging gender inequity through male involvement in maternal and newborn health: critical assessment of an emerging evidence base. Cult Health Sex.

