# Supplementary material for: Social determinants of male partner attendance in women’s prevention-of mother-to-child transmission program in Malawi
Source: BMC Public Health. 2020 Nov 30;20:1821. doi: 10.1186/s12889-020-09800-4 (PMC7708238; doi:10.1186/s12889-020-09800-4)
Supplement: Supplementary file 1 — Additional file 1. Knowledge on HIV/AIDS transmission (1–8), prevention and treatment (9–15) among women accompanied (n = 82) and not accompanied by the male partner (n = 46). [file 12889_2020_9800_MOESM1_ESM.docx]

**Additional File 1.** Correct Knowledge on HIV/AIDS transmission (1-8), prevention and treatment (9-15) among women accompanied (*n*=82) and not accompanied (n=46) by male partners.

| **Statement** | **All, n (%)** | **Women accompanied by male partner, n (%)** | **Women not accompanied by male partner, n (%)** | **P-value*** |
| --- | --- | --- | --- | --- |
| 1. HIV can be transmitted from mother to child during pregnancy | 117 (91.4) | 72 (87.8) | 45 (97.8) | 0.053 |
| **2. HIV can be transmitted from mother to child during breastfeeding** | **116 (90.6)** | **71 (86.6)** | **45 (97.8)** | **0.036** |
| 3. HIV can be transmitted by eating and drinking from the same plate or glass of an HIV-positive person | 108 (84.4) | 70 (85.4) | 38 (82.6) | 0.706 |
| 4. HIV can be transmitted by unprotected (no condom) sexual intercourse | 103 (80.5) | 70 (85.4) | 33 (71.7) | 0.057 |
| **5. HIV can be transmitted by mosquito/insect bite** | **108 (84.4)** | **65 (79.3)** | **43 (93.5)** | **0.033** |
| 6. HIV can be transmitted by kissing | 114 (89.1) | 74 (90.2) | 40 (87) | 0.576 |
| 7. HIV can be transmitted by contacting sneeze, cough or saliva of PLHIV | 111 (86.7) | 68 (82.9) | 43 (93.5) | 0.105 |
| 8.  HIV can be transmitted by specific socio-cultural practices, such as initiation ceremonies, widow inheritance or offering of ‘dry sex’ to please the partner | 123 (96.1) | 79 (96.3) | 44 (95.7) | 0.859 |
| 9. HIV can be prevented by properly using condom during sexual intercourse | 121 (94.5) | 78 (95.2) | 43 (93.5) | 0.696 |
| **10. HIV transmission can be avoided by remaining faithful to a single partner** | **124 (96.9)** | **82 (100)** | **42 (91.3)** | **0.007** |
| **11. A healthy-looking person can have HIV infection** | **97 (75.8)** | **68 (82.9)** | **29 (63)** | **0.012** |
| 12. An HIV-infected male can be cured of HIV if he has sex with a young girl who is a virgin (a girl who has never had sex before) | 112 (87.5) | 69 (84.2) | 43 (93.5) | 0.170 |
| **13. Early diagnosis/treatment increases PLHIV’s life expectancy** | **92 (71.9)** | **68 (82.9)** | **24 (52.2)** | **0.002** |
| **14. Therapy allows you to improve your physical status** | **92 (71.9)** | **68 (82.9)** | **24 (52.2)** | **0.002** |
| 15. HIV can be treated by traditional healer or a prophet | 115 (89.8) | 74 (90.2) | 41 (89.1) | 0.834 |

* Mann-Whitney test
