# Supplementary material for: Social determinants of male partner attendance in women’s prevention-of mother-to-child transmission program in Malawi
Source: BMC Public Health. 2020 Nov 30;20:1821. doi: 10.1186/s12889-020-09800-4 (PMC7708238; doi:10.1186/s12889-020-09800-4)
Supplement: Supplementary file 2 — Additional file 2. Positive attitude toward people living with HIV/AIDS among women accompanied (n = 82) and not accompanied by the male partner (n = 46). [file 12889_2020_9800_MOESM2_ESM.docx]

**Additional File 2.** Positive attitude toward people living with HIV/AIDS among women accompanied (*n*=82) and not accompanied (n=46) by male partners.

| **Statement** | **All, n (%)** | **Women accompanied by male partner, n (%)** | **Women not accompanied by male partner, n (%)** | **P-value*** |
| --- | --- | --- | --- | --- |
| 1. If one of your relative, who is HIV positive, becomes ill, would you be willing to care for her/him in your house or community? | 128 (100.0) | 82 (100.0) | 46 (100.0) | . |
| 2. If your friend is HIV positive, would you continue your friendship with him/her? | 127 (99.2) | 81 (98.8) | 46 (100.0) | 0.454 |
| 3. HIV is a punishment for the sins and immoralities of the past | 53 (41.4) | 39 (47.6) | 14 (30.4) | 0.568 |
| 4. I prefer to break my contacts with PLHIV | 114 (89.1) | 74 (90.2) | 40 (87) | 0.601 |
| 5. PLHIV brings disgrace and shame of their family | 104 (81.3) | 65 (82.0) | 39 (84.8) | 0.401 |
| 6. I can share a table PLHIV | 94 (73.4) | 63 (76.8) | 31 (67.4) | 0.248 |
| 7. If I become infected with HIV, my life will be over | 116 (90.6) | 75 (91.5) | 41 (89.1) | 0.687 |

* Mann-Whitney test
