# Supplementary material for: Social determinants of male partner attendance in women’s prevention-of mother-to-child transmission program in Malawi
Source: BMC Public Health. 2020 Nov 30;20:1821. doi: 10.1186/s12889-020-09800-4 (PMC7708238; doi:10.1186/s12889-020-09800-4)
Supplement: Supplementary file 3 — Additional file 3. Safe practice toward HIV/AIDS among women accompanied (N = 82) and not accompanied by the male partner (n = 46). [file 12889_2020_9800_MOESM3_ESM.docx]

**Additional File 3****.** Safe practice toward HIV/AIDS among women accompanied (*n*=82) and not accompanied (n=46) by male partners.

| **Statement** | **All, n (%)** | **Women accompanied by male partner, n (%)** | **Women not accompanied by male partner, n (%)** | **P-value*** |
| --- | --- | --- | --- | --- |
| 1. I did HIV test in the past | 72 (56.3) | 50 (61.0) | 22 (47.8) | 0.148 |
| 2. I used a condom during last 3 sexual intercourse | 29 (22.7) | 22 (26.8) | 7 (15.2) | 0.117 |
| 3. Have you ever had extramarital sex? | 97 (75.8) | 60 (73.2) | 37 (80.4) | 0.361 |
| 4. Do you use alcohol or drugs during/before sex? | 118 (92.2) | 74 (90.2) | 44 (95.7) | 0.291 |
| 5. You went to a traditional healer before going here | 120 (93.8) | 77 (93.9) | 43 (93.5) | 0.934 |

 *Mann-Whitney test
