# Supplementary material for: Social determinants of male partner attendance in women’s prevention-of mother-to-child transmission program in Malawi
Source: BMC Public Health. 2020 Nov 30;20:1821. doi: 10.1186/s12889-020-09800-4 (PMC7708238; doi:10.1186/s12889-020-09800-4)
Supplement: Supplementary file 4 — Additional file 4. Median of correct knowledge, positive attitude and safe practice toward HIV/AIDS among women accompanied (n = 82) and not accompanied by the male partner (n = 46). [file 12889_2020_9800_MOESM4_ESM.docx]

**Additional File 4.** Median scores of knowledge, attitude and practice toward HIV/AIDS among accompanied (*n*=82) and not accompanied (n=46) by male partners.

| **KAP** | **Median** | **Women accompanied by male partner** | **Women not accompanied by male partner** | **P-value*** |
| --- | --- | --- | --- | --- |
| Knowledge score | 86.7 | 93.3 | 83.3 | 0.133 |
| Attitude score | 85.7 | 85.7 | 85.7 | 0.187 |
| Practice score | 60.0 | 60.0 | 60.0 | 0.623 |

* Mann-Whitney test
