# Supplementary material for: Social determinants of male partner attendance in women’s prevention-of mother-to-child transmission program in Malawi
Source: BMC Public Health. 2020 Nov 30;20:1821. doi: 10.1186/s12889-020-09800-4 (PMC7708238; doi:10.1186/s12889-020-09800-4)
Supplement: Supplementary file 5 — Additional file 5 Knowledge, attitude and practice toward HIV of women in PMTCT in Malawi (n = 128)*. *A different scoring system was applied (score of ‘1’ for each correct, ‘0’ for each uncertain, ‘-1’ for each wrong answers). [file 12889_2020_9800_MOESM5_ESM.docx]

**Additional File 5.** Knowledge, attitude and practice toward HIV among women accompanied (n=82) and not accompanied (n=46) by male partners. *

| **KAP Survey** | **All, n (%)** | **Women accompanied by the male partners, n (%)** | **Women not accompanied by the male partners, n (%)** | **p-value** |
| --- | --- | --- | --- | --- |
| **Knowledge** |  |  |  | 0.057 |
| Low | 50 (39.1) | 27 (32.9) | 23 (50.0) |  |
| High | 78 (60.9) | 55 (67.1) | 23 (50.0) |  |
| **Attitude** |  |  |  | 0.071 |
| Negative | 48 (37.5) | 26 (31.7) | 22 (47.8) |  |
| Positive | 80 (62.5) | 56 (68.3) | 24 (52.2) |  |
| **Practice** |  |  |  | 0.836 |
| Risky | 66 (51.6) | 41 (50.0) | 25 (54.3) |  |
| Safe | 62 (48.4) | 41 (50.0) | 21 (45.7) |  |
| **Total** | 128 (100.0) | 82 (100.0) | 46 (100.0) |  |

* A different scoring system was applied (score of ‘1’ for each correct, ‘0’ for each uncertain, ‘-1’ for each wrong answer)
