# Supplementary material for: Social determinants of male partner attendance in women’s prevention-of mother-to-child transmission program in Malawi
Source: BMC Public Health. 2020 Nov 30;20:1821. doi: 10.1186/s12889-020-09800-4 (PMC7708238; doi:10.1186/s12889-020-09800-4)
Supplement: Supplementary file 6 — Additional file 6. Univariable and multivariable analysis examining association between male attendance and explanatory variables (n = 128)*. *A different scoring system was applied (score of ‘1’ for each correct, ‘0’ for each uncertain, ‘-1’ for each wrong answers). [file 12889_2020_9800_MOESM6_ESM.docx]

**Additional File 6.** Univariable and multivariable analysis examining association between male attendance and explanatory variables (n=128)*

|  | | **Crude OR** | | | **Adjusted OR** |  |
| --- | --- | --- | --- | --- | --- | --- |
|  | | Estimate (95% CI) | | | Estimate (95% CI) |  |
| **Socio-demographic** **variables** | |  | | |  |  |
| Age (years) | | 0.97 (0.91- 1.03) | | | 0.97 (0.90- 1.04) |  |
| Education | |  | | |  |  |
| No education/primary | | 1 | | | 1 |  |
| Secondary/Pre-University | | 0.92 (0.44-1.91) | | | 0.79 (0.34-1.82) |  |
| Employment | |  | | |  |  |
| Employed | | **1** | | | **1** |  |
| Unemployed | | **0.37 (0.16-0.84)** | | | **0.35 (0.14-0.89)** |  |
| Owning a means of transport | |  | | |  |  |
| No | | **1** | | | **1** |  |
| Yes | | **0.20 (0.07-0.64)** | | | **0.24 (0.07-0.80)** |  |
| Travel time to the facility (minutes) | |  | | |  |  |
| 0-89 | | 1 | | |  |  |
| >89 | | 2.21 (0.90- 5.42) | | |  |  |
| Means of transport | |  | | |  |  |
| Minibus, motorbike, car | | 1 | |  | | |
| Bike, by foot, other | | 0.66 (0.19-2.31) | |  | | |
| Electricity available in the dwelling | |  | | |  |  |
| No | | 1 | | |  |  |
| Yes | | 0.60 (0.29- 1.24) | | |  |  |
| **Knowledge, attitude and practice toward HIV** | |  | | |  |  |
| Level of Knowledge | |  | | |  |  |
| Low | | | 1 | | 1 | |
| High | | | 2.04 (0.97-4.26) | | 1.86 (0.76-4.55) | |
| Level of Attitude | |  | | |  |  |
| Negative | | 1 | | | 1 |  |
| Positive | | 1.97 (0.94- 4.15) | | | 1.39 (0.57-3.44) |  |
| Level of Practice | |  | | |  |  |
| Risky | | 1 | | | 1 |  |
| Safe | | 1.19 (0.58 – 2.45) | | | 1.12 (0.50-2.55) |  |

* A different scoring system was applied (score of ‘1’ for each correct, ‘0’ for each uncertain, ‘-1’ for each wrong answer)
